# Pubescenosides E–K, Seven New Triterpenoid Saponins from the Roots of *Ilex pubescens* and Their Anti-Inflammatory Activity

**DOI:** 10.3390/molecules23061426

**Published:** 2018-06-12

**Authors:** Xiaoxu Qiao, Mengying Ji, Yunda Yao, Leilei Ma, Jinjun Wu, Guochao Liao, Hua Zhou, Zhongqiu Liu, Peng Wu

**Affiliations:** 1 Joint Laboratory for Translational Cancer Research of Chinese Medicine of the Ministry of Education of the People’s Republic of China, International Institute for Translational Chinese Medicine, University of Chinese Medicine, Guangzhou 510006, China; qiaoxiaoxu87@126.com (X.Q.); 18826415793@163.com (M.J.); maleilei1@126.com (L.M.); wujinjun@gzucm.edu.cn (J.W.); liao@gzucm.edu.cn (G.L.); liuzq@gzucm.edu.cn (Z.L.); 2 State Key Laboratory of Quality Research in Chinese Medicine, Macau University of Science and Technology, Taipa, Macau 999078, China; Yunda_Yao@126.com (Y.Y.); huazhou2009@gmail.com (H.Z.)

**Keywords:** *Ilex pubescens*, anti-inflammation, RAW264.7

## Abstract

Seven new triterpenoid saponins (**1**–**7**), together with three known ones (**8**–**10**), were isolated from *Ilex pubescens*. Elucidation of their structures was performed based on high-resolution electrospray ionisation mass spectrometry (HR-ESI-MS), infrared spectra (IR), and nuclear magnetic resonance (NMR) spectroscopic data. The anti-inflammatory activity of the isolates toward lipopolysaccharide (LPS)-stimulated RAW264.7 macrophages was investigated. The results demonstrated that compounds **3**, **5**, and **6** inhibited the expression of inducible nitric oxide synthase (iNOS) protein in comparison with LPS stimulation in RAW264.7 cells.

## 1. Introduction

*Ilex pubescens*, belonging to the plant family Aquifoliaceae, is widely distributed throughout the south of China. The roots and leaves of *Ilex pubescens* have been used in folk medicine for treating thromboangiitis obliterans [1], coronary heart disease [2], and peripheral vascular diseases [3]. The primary components that have been reported from this plant include triterpenoids [4], phenolic glycosides [5], lignan glycosides [6], hemiterpene glycosides [7], and flavonoids [8]. All of the components described above play an important role related to several bioactivities, such as anti-inflammatory [9,10,11,12], anticoagulant [13], and antithrombotic activities [14].

Lipopolysaccharide (LPS)-induced macrophages are widely used to study inflammatory responses in vitro [15]. Macrophages are versatile cells that play many roles, and can be activated by external stimuli, such as LPS, to release excessive amounts of inflammatory mediators, including prostaglandin E_2_ (PGE_2_), cyclooxygenase-2 (COX-2), and inducible nitric oxide synthase (iNOS) [16,17]. Moreover, iNOS and COX-2 are considered to be the most important inflammatory mediators [16,18]. In our preliminary research, 27 triterpenoid saponins were isolated from *Ilex pubescens*, and some of them showed remarkable anti-inflammatory activity [19]. However, whether other compounds from this plant have anti-inflammatory activity is still unclear. Therefore, we used an LPS-induced macrophage inflammatory model to determine the effect of 10 compounds on the expression levels of iNOS and COX-2 protein.

## 2. Results

### 2.1. Characterization of the Compounds

**Figure 1:**
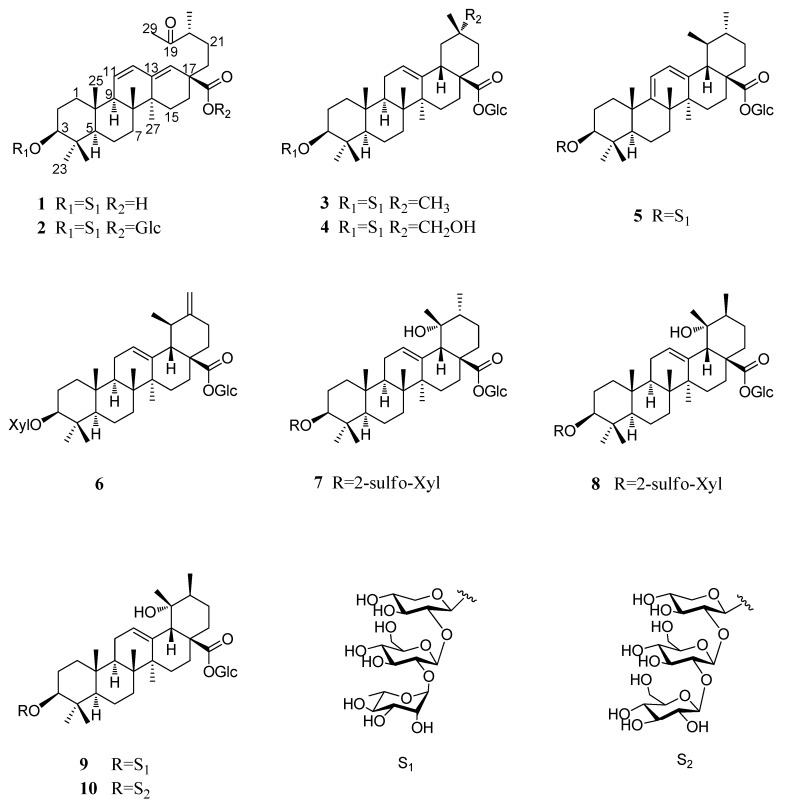
Structures of compounds **1**–**10**.

**Figure 2:**
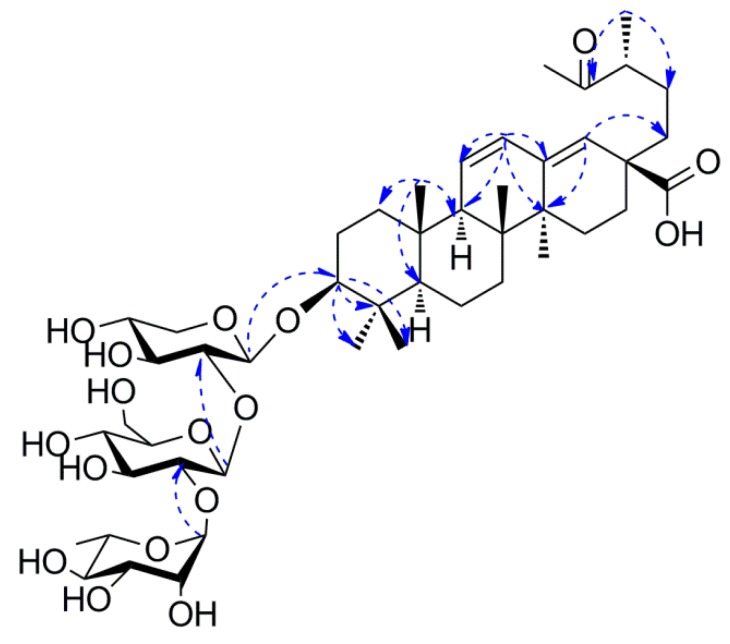
Key HMBC correlations of compound **1**.

**Figure 3:**
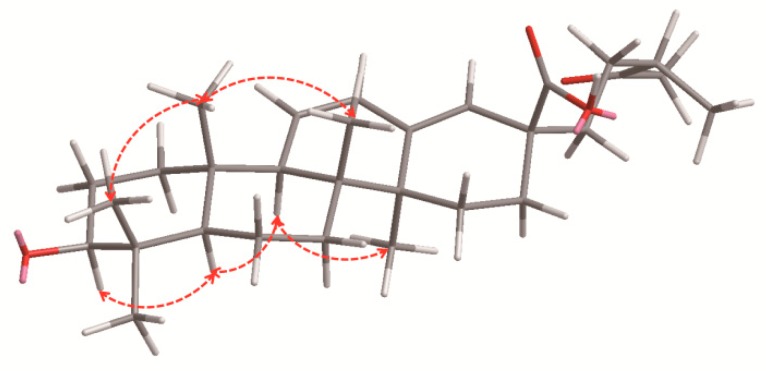
Key ROESY correlations of compound **1**.

Pubescenoside **E** (**1**) (Figure 1) was isolated as a white amorphous powder, and the molecular formula was deduced to be C_47_H_74_O_17_ based on the quasi-molecular ion peak [M + COOH]^−^ at *m*/*z* 955.4910 (calcd. 955.4908) in the negative-ion HR-ESI-MS (Figure S1, see Supplementary Materials). The sugar component of acid-hydrolyzed **1** gave xylose, glucose, and rhamnose. The glucose and xylose were determined to be d-configuration and the rhamnose be l-configuration, via thin-layer chromatography (TLC) and high-performance liquid chromatography (HPLC) analyses. The IR spectrum demonstrated the presence of hydroxyl (3426 cm^−1^), alkyl (2938 cm^−1^), carbonyl (1703 cm^−1^), and double bond (1644 cm^−1^) groups. The ^1^H NMR and ^13^C NMR (Table 1 and Table 2) showed 17 carbon signals for three sugar moieties and 30 carbons for the aglycone, including one ketone group at δ_C_ 212.0 (C-19); one di-substituted double bond (*δ*_H_ 5.62, *δ*_C_ 127.7, C-11; *δ*_H_ 6.16, *δ*_C_ 130.8, C-12); one tri-substituted double bond (*δ*_C_ 142.5, C-13; *δ*_H_ 5.88, *δ*_C_ 129.6, C-18); one carboxyl (*δ*_C_ 178.9, COOR-28); six singlets for tertiary methyls at *δ*_H_ 0.82, 0.85, 1.06, 1.10, 1.34 and 2.21; one methyl doublet at *δ*_H_ 1.08; and anomeric protons of three sugar units (*δ*_H_ 4.91, *δ*_C_ 106.2; *δ*_H_ 5.79, *δ*_C_ 102.6; *δ*_H_ 6.39, *δ*_C_ 102.3). When compared with 3*β*-hydroxy-19-oxo-18,19-seco-11,13(18)-ursa-diene-28-oic acid [20], their structures were very similar, except for the additional sugar units at C-3 in **1**. The heteronuclear multiple bond correlation (HMBC) analysis results were as follows: from H-3 to C-4, C-23, C-24, and inner-Xyl-C-1; from H-12 to C-9, C-11, C-13, and C-14; from H-18 to C-14, C-16, C-17, and C-22; and from CH_3_-30 to C-19, C-20, and C-21. The HMBC from terminal-Rha-H-1 (*δ*_H_ 6.39, s) to the intermediate-Glc-C-2 (*δ*_C_ 79.7), and from the intermediate-Glc-H-1 (*δ*_H_ 5.79, d, *J* = 5.8 Hz) to the inner-Xyl-C-2 (*δ*_C_ 79.3), established the linkages of the sugar moieties (Figure 2). The rotating frame overhauser effect spectroscopy (ROESY) correlations of H-3/H-5, H-5/H-9, and H-9/Me-27 revealed Me-27 to be *α*-oriented, and the correlations of Me-23/Me-25 and Me-25/Me-26 indicated that the sugar moieties of C-3, Me-23, Me-25, and Me-26 were determined to be in *β*-orientations (Figure 3). Along with further analyses of the ^1^H-^1^H correlation spectroscopy (^1^H-^1^H COSY), heteronuclear single quantum coherence (HSQC), and HMBC spectra, compound **1** was finally identified as 3*β*-[*α*-l-rhamnopyranosyl-(1→2)-*β*-d-glucopyranosyl-(1→2)-*β*-d-xylopyranosyl]-urs-19-oxo-18,19-secoursa-11,13(18)-dien-28-oic acid (Figure 1).

The molecular formula of Pubescenoside **F** (**2**), a white amorphous powder, was determined to be C_53_H_84_O_22_ by the HRESIMS ion at *m*/*z* 1071.5388 [M − H]^−^ (calcd. 1071.5381) and NMR data. The IR spectrum revealed the existence of hydroxyl, olefinic, and carboxyl absorption bands. The sugar components of acid-hydrolyzed **2** included d-Xylose, d-glucoses, and l-rhamnose, as identified by TLC and HPLC analyses. The ^13^C NMR spectrum (Table 2) showed 53 carbon signals, including 23 carbon signals belonging to the sugar units and 30 carbon signals belonging to the aglycone part. It also revealed one ketone group at *δ*_C_ 212.3 (C-19), one carboxyl at *δ*_C_ 175.1 (C-28), and four anomeric carbons. The ^1^H NMR spectrum (Table 1) of **2** displayed signals assignable to six angular methyl groups at *δ*_H_ 0.76, 0.82, 1.03 (6H), 1.32, and 2.13 and one methyl doublet at *δ*_H_ 1.04. The structure of **2** resembled that of **1**, except for an additional glucose unit at C-28. The HMBC from Glc-H-1 to C-28 and inner-Xyl-H-1 to C-3 demonstrated glycosylation sites at the 28-*O*- and 3-*O*- positions. Eventually, compound **2** was elucidated as *β*-d-glucopyranosyl 3*β*-[*α*-l-rhamnopyranosyl-(1→2)-*β*-d-glucopyranosyl-(1→2)-*β*-d-xylopyranosyl]-urs-19-oxo-18,19-secoursa-11,13(18)-dien-28-oate (Figure 1).

The HR-ESI-MS of Pubescenoside **G** (**3**) displayed a molecular ion peak [M + COOH]^−^ at *m*/*z* 1103.5642 (calcd. 1103.5644), indicating that its molecular formula was C_53_H_86_O_21_. The IR data also manifested absorption bands for hydroxyl, alkyl, carbonyl, and double bond groups. The ^1^H NMR and ^13^C NMR data (Table 1 and Table 2) of compound **3** demonstrated 23 carbon signals for sugar moieties and 30 carbons for the aglycone, including one tri-substituted double bond (*δ*_H_ 5.41, *δ*_C_ 123.1, C-12; *δ*_C_ 144.4, C-13), one ester carbonyl carbon at C-28 (*δ*_C_ 176.8), four anomeric carbon signals, and seven methyl singlets at *δ*_H_ 0.82, 0.87, 0.91, 1.07 (6H), 1.24, and 1.33. The NMR data of **3** and 3-*β*-*O*-{[*O*-*β*-d-galactopyranosyl-(1→3)-*β*-d-glucopyranosyl-(1→2)]-*β*-d-glucooyranosyl}oleanolicacid 28-*β*-d-glucopyranosyl ester [21] were highly similar, with the main difference being the type and connection of the sugar at C-3. The HMBC analysis results were as follows: from H-12 to C-9 and C-14; from H-24 to C-3, C-4, and C-5; from H-27 to C-13, C-14, and C-15; from H-29 to C-19, C-20, and C-21; from inner-Xyl-H-1 to C-3; and from the intermediate-Glc-H-1 to the inner-Xyl-C-2. When combined with comprehensive analyses of ^1^H-^1^H COSY, HMBC, HSQC, and NOESY NMR spectra, we identified the structure of compound **3** to be *β*-d-glucopyranosyl 3*β*-[*α*-l-rhamnopyranosyl-(1→2)-*β*-d-glucopyranosyl-(1→2)-*β*-d-xylopyranosyl]-olean-12-en-28-oate (Figure 1).

The HR-ESI-MS data ([M − H]^−^ at *m*/*z* 1073.5614, calcd. 1073.5538) indicated that the molecular formula of Pubescenoside **H** (**4**) was C_53_H_86_O_22_. The IR spectrum demonstrated the existence of hydroxyl, olefinic, and carboxyl absorption bands. The configurations of the sugar units were determined by hydrolysis to be d-Xylose, d-glucoses, and l-rhamnose. The ^1^H NMR and ^13^C NMR spectrum (Table 1 and Table 2) gave six methyl proton signals at *δ*_H_ 0.83, 1.08 (6H), 1.09, 1.25, and 1.32; one double bond (*δ*_H_ 5.45, *δ*_C_ 123.1,C-12; *δ*_C_ 144.6, C-13); one quaternary carbon at C-29 (*δ*_C_ 74.0); one ester carbonyl carbon at C-28 (*δ*_C_ 176.8); and anomeric protons of the four sugar units (3-*O*-inner-Xyl-1, *δ*_H_ 4.88, *δ*_C_ 106.1; intermediate-Glc-1, *δ*_H_ 5.82, *δ*_C_ 102.5; terminal-Rha-1, *δ*_H_ 6.40, *δ*_C_ 102.3; 28-*O*-Glc-1, *δ*_H_ 6.33, *δ*_C_ 96.0). The NMR data for compound **4** were almost same as for compound **3**, except for the additional hydroxyl group at C-29. The linkages were confirmed by observation of HMBC from H-29 (*δ*_H_ 3.56) to C-19 (*δ*_C_ 41.2), C-20 (*δ*_C_ 36.7), and C-21 (*δ*_C_ 29.1). Based on these results, the structure of **4** was established as *β*-d-glucopyranosyl 3*β*-[*α*-l-rhamnopyranosyl-(1→2)-*β*-d-glucopyranosyl-(1→2)-*β*-d-xylopyranosyl]-29*α*-hydroxyurs-olean-12-en-28-oate (Figure 1).

Pubescenoside **I** (**5**) was obtained as a white amorphous powder with the molecular formula C_53_H_84_O_21_ (HR-ESI-MS: *m*/*z* 1101.5488 [M + COOH]^−^). In the IR spectrum, absorption bands for hydroxyl (3396 cm^−1^), alkyl (2930 cm^−1^), carbonyl (1729 cm^−1^), and double bond groups (1641 cm^−1^) were observed. The configuration of the sugar units was ascertained by hydrolysis to be d-Xylose, d-glucoses, and l-rhamnose. The ^13^C NMR data (Table 2) indicated that compound **5** had 53 carbon signals, containing 30 carbon signals in the aglycone and 23 carbon signals in the sugar unit. The 1D and 2D NMR spectra (Table 1 and Table 2) revealed the presence of one tri-substituted double bond (*δ*_C_ 134.6, C-9; *δ*_H_ 6.58, *δ*_C_ 128.6, C-11); another tri-substituted double bond (*δ*_H_ 5.61, *δ*_C_ 125.8, C-12; *δ*_C_ 138.6, C-13); one ester carbonyl carbon at C-28 (*δ*_C_ 176.5); four anomeric signals (*δ*_H_ 4.84, *δ*_C_ 106.1; *δ*_H_ 5.71, *δ*_C_ 102.2; *δ*_H_ 6.30, *δ*_C_ 102.1; *δ*_H_ 6.23, *δ*_C_ 96.2); five tertiary methyl at *δ*_H_ 0.81, 0.98, 0.99, 1.04, and 1.27 and two methyl doublets at *δ*_H_ 1.32 (d, *J* = 7.0 Hz) and *δ*_H_ 0.76 (d, *J* = 5.9 Hz). The results described above indicated that Pubescenoside **I** (**5)** was highly similar to ilexsaponion **L** [19], except for an additional sugar unit. The HMBC correlations from terminal-Rha-H-1 (*δ*_H_ 6.30, s) to the intermediate-Glc-C-2 (*δ*_C_ 79.5) and from the intermediate-Glc-H-1 (*δ*_H_ 5.71, d, *J* = 6.8 Hz) to the inner-Xyl-C-2 (*δ*_C_ 79.0), established the linkages of the sugar moieties. Finally, we identified the structure as *β*-d-glucopyranosyl 3*β*-[*α*-l-rhamnopyranosyl-(1→2)-*β*-d-glucopyranosyl-(1→2)-*β*-d-xylopyranosyl]-urs-9(11),12-dien-28-oate (Figure 1).

Pubescenoside J (**6**) was obtained as white amorphous powder and had a molecular formula of C_41_H_64_O_12_, deduced from an ion peak in the HR-ESI-MS at *m/z* 793.4462 [M + COOH]^−^ (calcd. for C_42_H_64_O_14_^−^, 793.4380). The IR spectrum of **6** showed hydroxyl, alkyl, and carbonyl moieties at 3385 cm^−1^, 2941 cm^−1^, and 1729 cm^−1^, respectively. The sugar components of acid-hydrolyzed **6** included d-xylose and d-glucose, as identified through TLC and HPLC analyses. The ^1^H NMR and ^13^C NMR spectrum (Table 1 and Table 2) of the aglycone of **6** revealed five singlets for tertiary methyls at *δ*_H_ 0.88, 0.99, 1.09, 1.27, and 1.31; one methyl doublet at *δ*_H_ 1.06 (d, *J* = 7.1 Hz); one carboxylic acid (*δ*_C_ 176.5, COOH-28); and two anomeric signals (*δ*_H_ 4.86, *δ*_C_ 108.0,CH-Xyl-1; *δ*_H_ 6.34, *δ*_C_ 96.3, CH-Glc-1). The HMBC analysis was as follows: from H-12 to C-9, C-11 and C-14; from H-18 to C-12, C-13, C-14, C-16, C-17, C-20, C-28 and C-30; from H-25 to C-1, C-5, and C-9; and from H-30 to C-18, C-19 and C-20. A precise comparison of its ^1^H and ^13^C NMR data with those of ilexsaponin I [19] indicated structural similarity, except for an additional sugar unit in ilexsaponin I. Finally, **6** was elucidated as *β*-d-glucopyranosyl 3*β*-*β*-d-xylopyranosyl-urs-12,20(30)-dien-28-oate (Figure 1).

The molecular formula of Pubescenoside **K** (**7**) was inferred from the HR-ESI-MS(negative ion mode) result, which displayed [M − H]^−^ ions at *m*/*z* 845.4052 (calcd. for C_41_H_66_O_16_S-H = 845.3999). The IR spectrum also showed absorption signals for hydroxyl, double bond, and ester groups. The ^1^H NMR data (Table 1) of **7** showed six singlets for tertiary methyls at *δ*_H_ 0.89, 1.14, 1.18, 1.42 (6H), and 1.73, and one methyl doublet at *δ*_H_ 1.11. Furthermore, signals for one tri-substituted double bond (*δ*_H_ 5.57, *δ*_C_ 128.4, C-12; *δ*_C_ 139.3, C-13), one ester carbonyl carbon at C-28 (*δ*_C_ 177.3), one xylopyranose linked to C-3 of the aglycone, and one *β*-d-glucopyranose linked to C-28 of the aglycone were observed. The HMBC analysis results were as follows: from H-12 to C-9, C-14, and C-18; from H-18 to C-16, C-17, C-19, and C-20; from H-23 to C-3, C-4 and C-5; from H-25 to C-1, C-5, C-9 and C-10; from H-30 to C-19, C-20, and C-21; and from inner-Xyl-H-1 to C-3. The difference between compound **7** and ilexpublesnin **E** [4] was that compound **7** had sulfonylation on the hydroxyl attached to Xyl-C-2, whereas ilexpublesnin **E** is connected to three sugar units at C-3. Therefore, based on the above analysis, compound **7** was deduced as *β*-d-glucopyranosyl 3*β-*[(2-*O*-sulfo-*β*-d-xylopyranosyl)oxy]-19*α*-hydroxy-urs-12-en-28-oate (Figure 1).

Additionally, three known compounds (**8**–**10**) (Figure 1) were also isolated, and their structures were identified as ilexpublesnin **I** (**8**) [4], ilexoside **O** (**9**) [2], and ilexpublesnin **J** (**10**) [4], by comparison of their ^1^H and ^13^C NMR, as well as MS data with reported values.

### 2.2. Anti-Inflammatory Activity

**Figure 4:**
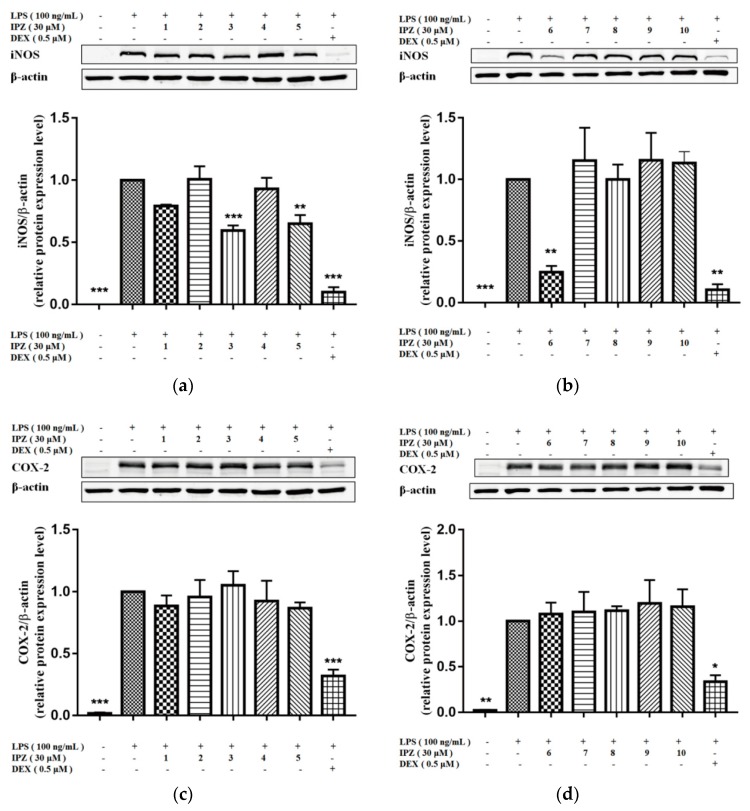
Effect of compounds **1**–**10** (IPZ 1-10) on inducible nitric oxide synthase (iNOS) and cyclooxygenase-2 (COX-2) protein expression in lipopolysaccharide (LPS)-stimulated RAW264.7 cells. RAW264.7 cells were seeded in 24-well plate and cultured overnight. Subsequently, compounds **1**–**10** and the positive control dexamethasone (DEX) were administered to RAW264.7 cells for 1 h, and LPS (100 ng/mL) was used to stimulate the cells for the final 18 h. Western blotting was performed to analyze protein expression of iNOS, COX-2, and the corresponding values were quantitated by Odyssey v3.0 software. The results are expressed as the mean ± SEM, *n* = 3. * *p* < 0.05, ** *p* < 0.01, *** *p* < 0.001, vs. LPS group.

The anti-inflammatory activity of compounds **1**–**10** was evaluated by utilizing a LPS-stimulated RAW264.7 cell model. As presented in Figure 4, when compared with LPS stimulation in RAW264.7 cells, compounds **3**, **5**, and **6** exhibited inhibitory effects on the expression of iNOS protein, and the positive control drug dexamethasone (DEX) notably inhibited the expression of iNOS and COX-2 protein. The effects of DEX intervention suggested that the experimental procedure was adequate.

## 3. Discussion

In summary, seven new triterpenoid saponins, named Pubescenosides **E**–**K**, together with three known ones, were isolated from the roots of *Ilex pubescens*. Elucidation of their structures was performed based on extensive spectroscopic analyses. The anti-inflammatory activity of the isolates toward lipopolysaccharide (LPS)-stimulated RAW264.7 macrophages was investigated. The results demonstrated that compounds **3**, **5**, and **6** inhibited iNOS protein expression in LPS-stimulated RAW264.7 cells with dexamethasone as a positive control. The findings revealed that compounds **3**, **5**, and **6** might have anti-inflammatory activity and might have potential value in anti-inflammatory treatments.

## 4. Materials and Methods 

### 4.1. General Experimental Procedures

A Jasco P-1020 digital polarimeter (Jasco, Tokyo, Japan) was used to measure the optical rotations at the sodium D line (589 nm). IR spectra were collected with a Jasco Fourier transform (FT)/IR-480 plus spectrophotometer (Jasco, Tokyo, Japan) for scanning the IR spectrum with KBr pellets. HRESIMS data were acquired on an Agilent 6540 Q-TOF mass spectrometer (Agilent Technologies, Palo Alto, CA, United States). The 1D and 2D NMR spectra were obtained using a Bruker AV-400 or a Bruker AV-600 spectrophotometer (Bruker, Faellanden, Switzerland), with tetramethylsilanein pyridine-*d*_5_ as an internal standard. Semi-preparative HPLC was carried out with a LC-6AD pump and SPD-M20A detector on an Inertsil PREP-ODS (10 μm, 20 × 250 mm) column (Gl Sciences Inc., Eindhoven, Netherlands). Silica gel (200–300 mesh, Qingdao Marine Chemical Plant, Qingdao, China), Sephadex LH-20 gel (25–100 μm, GE Healthcare, Biosciences AB, Uppsala, Sweden), and reverse phase C18 (50 μm, YMC, Kyoto, Japan) were used for column chromatography (CC). All reagents used were purchased from Tianjin Damao Chemical Company (Damao, Tianjin, China).

### 4.2. Plant Material

The roots of *Ilex pubescens* were collected near Conghua City, Guangdong Province, China, in July 2012, and identified by Prof. Guangxiong Zhou of the College of Pharmacy, Jinan University. The voucher specimen (12071002) is stored in the International Institute for Translational Chinese Medicine, Guangzhou University of Traditional Chinese Medicine, Guangzhou, China.

### 4.3. Extraction and Isolation

The powdered and dried roots of *I. pubescens* (40 kg) were extracted with 70% ethanol (2 × 320 L) at 70 °C. The extract was evaporated under vacuum to obtain 2.2 kg of a dark brown residue. The residue was separated on a D101 macroporous resin column with different proportions of EtOH–H_2_O (0:10, 3:7, 6:4, and 9:1), which yielded four fractions (Frs. 1 to 4). Fr. 3 (500 g) was loaded on a silica gel CC with CHCl_3_–CH_3_OH (99:1–1:1) eluent to yield 12 fractions (Frs. A to L). Compound **1** (20 mg), was isolated from Fr. K by ODS CC using MeOH and H_2_O and further purified through RP-C_18_ semi-preparative HPLC (MeOH–H_2_O 65:35; *t*_R_, 89.3 min). Fr. L (55 g) was separated into seven fractions (L_1_–L_7_) over silica gel CC with CH_2_Cl_2_–CH_3_OH (8:2–1:1) as the eluent. Fr. L_2_ (15.8 g) was chromatographically separated on ODS CC with MeOH–H_2_O (40:60–100:0), followed by a Sephadex LH-20 column (MeOH), and purified by semi-preparative HPLC to achieve compound **6** (15 mg, MeOH–H_2_O 76.5:23.5; *t*_R_, 35.5 min). Fr. L_3_ (17.5 g) was purified on ODS CC, eluting with MeOH–H_2_O (30:70–100:0) and further purified by semi-preparative HPLC to yield compound **2** (18 mg, MeOH–H_2_O 84:16; *t*_R_, 32 min); compound **3** (19 mg, MeOH–H_2_O 76:24; *t*_R_, 34.5 min); compound **5** (56 mg, MeOH–H_2_O 76:24; *t*_R_, 57 min); compound **7** (43 mg, CH_3_CN–H_2_O 22:78; *t*_R_, 32 min); compound **8** (32 mg, CH_3_CN–H_2_O 22:78; *t*_R_, 32 min); compound **9** (480 mg, MeOH–H_2_O 70:30; *t*_R_, 23 min); and compound **10** (10 mg, MeOH–H_2_O 76:24; *t*_R_, 20.5 min). Compound **4** (9.9 mg, MeOH–H_2_O, 62:28) was also obtained from Fr. L_4_ through the chromatographic methods described above. 

#### 4.3.1. Pubescenoside **E** (**1**)

White amorphous powder; [*α*]${}_{D}^{25.8}$ +10.74 (c 0.73 CH_3_OH); IR (KBr) *v*_max_: 3426, 2938, 2874, 1703, 1644, 1357, 1041 cm^−1^; NMR spectroscopic data (pyridine-*d*_5_, 400/100 MHz), see Table 1 and Table 2; HR-ESI-MS *m/z* 955.4910 [M + COOH]^−^ (calcd. for 955.4908).

#### 4.3.2. Pubescenoside **F** (**2**) 

White amorphous powder; [*α*]${}_{D}^{25.8}$ +3.52 (c 0.97 CH_3_OH); IR (KBr) *v*_max_: 3396, 2930, 2877, 1700, 1635, 1360, 1074 cm^−1^; NMR spectroscopic data (pyridine-*d*_5_, 600/150 MHz), see Table 1 and Table 2; HR-ESI-MS *m/z* 1071.5388 [M − H]^−^ (calcd. for 1071.5381).

#### 4.3.3. Pubescenoside **G** (**3**) 

White amorphous powder; [*α*]${}_{D}^{25.8}$ +13.9 (c 0.64 CH_3_OH); IR (KBr) *v*_max_: 3408, 2930, 2870, 1738, 1646, 1384, 1077 cm^−1^; NMR spectroscopic data (pyridine-*d*_5_, 600/150 MHz), see Table 1 and Table 2; HR-ESI-MS *m/z* 1103.5642 [M + COOH]^−^ (calcd. for 1103.5644)

#### 4.3.4. Pubescenoside **H** (**4**) 

White amorphous powder; [*α*]${}_{D}^{25.8}$ +14.6 (c 0.63 CH_3_OH); IR (KBr) *v*_max_: 3373, 2933, 2870, 1735, 1644, 1452, 1074 cm^−1^; NMR spectroscopic data (pyridine-*d*_5_, 600/150 MHz), see Table 1 and Table 2; HR-ESI-MS *m/z* 1073.5614 [M − H]^−^ (calcd. for 1073.5538).

#### 4.3.5. Pubescenoside **I** (**5**) 

White amorphous powder; [*α*]${}_{D}^{25.8}$ −5.2 (c 0.99 CH_3_OH); IR (KBr) *v*_max_: 3396, 2930, 2877, 1729, 1641, 1360, 1077 cm^−1^; NMR spectroscopic data (pyridine-*d*_5_, 600/150 MHz), see Table 1 and Table 2; HR-ESI-MS *m/z* 1101.5488 [M + COOH]^−^ (calcd. for 1101.5487). 

#### 4.3.6. Pubescenoside **J** (**6**) 

White amorphous powder; [*α*]${}_{D}^{25.8}$ +10.2 (c 0.53 CH_3_OH); IR (KBr) *v*_max_: 3385, 2941, 2874, 1729, 1074 cm^−1^; NMR spectroscopic data (pyridine-*d*_5_, 600/150 MHz), see Table 1 and Table 2; HR-ESI-MS *m/z* 793.4462 [M + COOH]^−^ (calcd. for 793.4380).

#### 4.3.7. Pubescenoside **K** (**7**) 

White amorphous powder; [*α*]${}_{D}^{25.8}$ +8.3 (c 0.89 CH_3_OH); IR (KBr) *v*_max_: 3458, 2938, 2880, 1735, 1646, 1230, 1065 cm^−1^; NMR spectroscopic data (pyridine-*d*_5_, 600/150 MHz), see Table 1 and Table 2; HR-ESI-MS *m/z* 845.4052 [M − H]^−^ (calcd. for 845.3999).

### 4.4. Acid Hydrolysis

Each solution of the seven new compounds (2 mg) was stirred in 2N HCl (5 mL) at 80 °C in a stoppered reaction vial for 5 h. The solution was evaporated to dryness under vacuum, and then the residue was compared with standard sugars by silica gel TLC. N-butanol-acetone-H_2_O (4:3:1) was chosen as the solvent system, and the spots were observed after spraying the plates with H_2_SO_4_ and heating at 105 °C for 1 min. The *R_f_* values of xylose, glucose, and rhamnose via TLC, which were hydrolyzed from Pubescenoside **E**–**K**, were 0.47, 0.60, and 0.71, respectively. The residue was dissolved in pyridine, and 2 mg of l-cysteine methyl ester hydrochloride was added. The mixture was stirred at 60 °C for 2 h, 5 μL *O*-tolyl isothiocyanate was added, and the mixture was stirred at 60 °C for another 2 h. After cooling, the reaction mixture was analyzed by reversed phase HPLC (RP-C18 column, λ = 250 nm, acetonitrile—0.1% formic acid 25:75, flow rate, 1.0 mL/min). The retention time of d-xylose (*t_R_*, 18.4 min), d-glucose (*t_R_*, 15.8 min), and l-rhamnose (*t_R_*, 26.2 min) was determined by comparison with standards.

### 4.5. Cell Culture and Western Blot

RAW264.7 cells were purchased from American Type Culture Collection (ATCC, Manassas, VA, USA) and cultured in Dulbecco’s Modified Eagle’s Medium containing 10% heat-inactivatedfetal bovine serum, penicillin G (100 units/mL), streptomycin (100 mg/mL), and l-glutamine (2 mM) in a humidified incubator containing 5% CO_2_ at 37 °C. Compounds **1**–**10** were completely dissolved in dimethyl sulfoxideto a final concentration of 30 mM, and the working concentration was 30 μΜ. RAW264.7 cells (8 × 10^4^ cells/wells) were seeded in 24-well plates for 24 h, pretreated with compounds **1**–**10** or dexamethasone for 1 h, and then stimulated with LPS (100 ng/mL) for another 18 h. Dexamethasone at a concentration of 0.5 μΜ was selected as a positive control for inhibition of iNOS and COX-2 expression. LPS-stimulated cells without any intervention were used as the model control, and cells cultured in DMEM medium were used as the normal control. The Western blot methods were based on our previous research [19].

## Figures and Tables

**Table 1:** ^1^H NMR spectroscopic data of compounds **1**–**7** (in pyridine-*d*_5_).

Position	1 ^a^	2 ^b^	3 ^b^	4 ^b^	5 ^b^	6 ^b^	7 ^b^
1	0.95 m; 1.77 m	0.94 m;1.71 m	0.95 m; 1.50 m	0.91 m; 1.46 m	0.97 m; 1.75 m	0.98 m; 1.48 m	0.90 m; 1.54 m
2	1.68 m; 2.56 m	1.75 m; 2.12 m	1.88 m; 2.06 m	1.86 m; 2.09 m	1.96 m; 2.13 m	1.89 m; 2.17 m	1.93 m; 2.12 m
3	3.30 dd (11.6, 3.7)	3.28 dd (11.5, 4.1)	3.28 dd (11.4, 3.6)	3.27 dd (11.4, 4.1)	3.27 dd (10.7, 3.1)	3.40 dd (11.5, 3.8)	3.29 dd (10.8, 2.5)
5	0.83 m	0.79 m	0.80 m	0.76 m	0.76 m	0.81 m	0.80 m
6	1.33 m; 1.54 m	1.27 m; 1.52 m	1.30 m; 1.49 m	1.25 m; 1.44 m	1.26 m; 1.47 m	1.27 m; 1.46 m	1.27 m; 1.45 m
7	1.33 m	1.26 m	1.46 m	1.31 m; 1.43 m	1.16 m	1.37 m; 1.46 m	1.40 m; 1.54 m
9	2.04 m	1.98 m	1.63 m	1.61 m	-	1.76 m	1.78 m
11	5.62 d (10.2)	5.59 d (9.8)	1.93 m	1.87 m	6.58 d (10.1)	1.91 m; 1.98 m	2.0 m; 2.06 m
12	6.18 d (8.5)	6.03 d (10.1)	5.42 s	5.45 s	5.61 d (10.6)	5.48 s	5.57 s
15	1.24 m; 1.92 m	1.16 m; 1.90 m	1.17 m; 2.33 m	1.15 m; 2.32 m	0.88 m; 1.93 m	1.17 m; 2.35 m	1.26 m; 2.44 m
16	1.23 m; 2.15 m	1.30 m; 2.13 m	1.17 m; 1.26 m	1.99 m; 2.16 m	1.64 m; 2.15 m	1.85 m; 1.99 m	3.12 m
18	5.88s	5.72 s	3.19 m	3.28 m	1.92 m	3.99 s	2.92 m
19	-	-	1.25 m; 1.75 m	1.40 m; 2.11 m	2.30 m	2.59 m	-
20	2.53 m	2.50 m	-	-	1.44 m	-	1.36 m
21	1.33 m; 1.66 m	1.32 m; 1.59 m	1.08 m; 1.33 m	1.24 m; 1.74 m	1.50 m; 1.58 m	1.28 m; 1.76 m	2.04 m; 2.12 m
22	1.99 m	1.95 m	1.75 m; 1.97 m	1.73 m; 1.84 m	1.28 m; 2.62 m	1.76 m; 2.08 m	1.79 m; 2.04 m
23	1.34 s	1.32 s	1.33 s	1.32 s	1.27 s	1.31 s	1.42 s
24	1.06 s	1.03 s	1.07 s	1.08 s	1.04 s	0.99 s	1.18 s
25	0.82 s	0.76 s	0.82 s	0.83 s	0.81 s	0.88 s	0.89 s
26	0.85 s	0.82 s	1.07 s	1.09 s	0.98 s	1.09 s	1.14 s
27	1.06 s	1.03 s	1.24 s	1.25 s	0.99 s	1.27 s	1.73 s
29	2.11 s	2.13 s	0.91 s	3.56 m	1.32 d (4.0)	1.06 d (6.7)	1.42 s
30	1.08 d (8.7)	1.03 d (7.1)	0.87 s	1.08 s	0.76 d (5.9)	5.04 ^c^	1.11 d (6.2)
3-*O*-	Xyl	Xyl	Xyl	Xyl	Xyl	Xyl	2-sulfo-Xyl
1	4.92 d (5.2)	4.89 d (6.7)	4.88 d (6.6)	4.88 d (6.6)	4.84 d (6.1)	4.85 d (7.4)	4.97 d (6.6)
2	4.42 ^e^	4.01 ^c^	4.29 ^d^	4.28 ^d^	4.22 ^d^	4.03 m	5.05 m
3	3.87 m	3.87 m	3.88 m	3.86 m	3.84 m	4.17 m	4.43 m
4	4.08 ^c^	4.06 ^c^	4.08 ^c^	4.06 ^c^	4.02 ^c^	4.21 m	4.21 m
5	3.73 m; 4.28 ^d^	3.72 m; 4.28 ^d^	3.72 m; 4.28 ^d^	3.72 m; 4.27 ^d^	3.68 m; 4.25 ^d^	3.78 m; 4.38 m	3.74 m; 4.21 m
Intermediate	Glc	Glc	Glc	Glc	Glc		
1	5.79 d (5.8)	5.80 d (7.3)	5.81 d (7.8)	5.82 d (7.2)	5.71 d (6.8)		
2	4.24 ^d^	4.27 ^d^	4.24 ^d^	4.22 ^d^	4.22 ^d^		
3	4.48 ^e^	4.44 ^e^	4.44 ^c^	4.41 ^e^	4.39 ^e^		
4	4.07 ^c^	4.06 ^c^	4.04 ^c^	4.03 ^c^	3.83 m		
5	4.28 ^d^	4.27 ^d^	4.43 ^e^	4.29 ^d^	4.39 ^e^		
6	4.27 ^d^; 4.50 ^e^	4.28 ^d^; 4.51 ^e^	4.28 ^d^; 4.51 ^e^	4.27 ^d^; 4.48 ^e^	4.21 ^d^; 4.48 ^e^		
Terminal	Rha	Rha	Rha	Rha	Rha		
1	6.39 br s	6.38 br s	6.39 br s	6.40 br s	6.30 br s		
2	4.71 m	4.71 m	4.75 m	4.76 m	4.69 s		
3	4.04 ^c^	4.05 ^c^	4.04 ^c^	4.04 ^c^	3.95 ^c^		
4	4.33 ^d^	4.34 ^d^	4.34 ^e^	4.23 ^d^	4.29 ^d^		
5	5.03 m	5.04 m	5.04 m	5.04 m	4.98 m		
6	1.81 ^f^	1.78 ^f^	1.79 d (6.0)	1.8 d (6.0)	1.75 d (5.5)		
28-*O*-		Glc	Glc	Glc	Glc	Glc	Glc
1		6.32 d (8.2)	6.32 d (8.4)	6.35 d (7.8)	6.23 d (7.8)	6.34 d (8.1)	6.25 d (7.8)
2		4.48 ^e^	4.21 ^d^	4.33 ^e^	4.13 ^d^	4.23 m	4.24 m
3		4.44 ^e^	4.29 ^d^	4.41 ^e^	4.39 ^e^	4.04 m	4.33 ^c^
4		4.29 ^d^	4.35 ^e^	4.35 ^e^	4.23 ^d^	4.37 m	4.33 ^c^
5		4.44 ^e^	4.29 ^d^	4.28 ^d^	4.22 ^d^	4.30 m	4.08 m
6		4.29 ^d^	4.43 ^e^	4.39 ^e^; 4.45 ^e^	4.27 ^d^; 4.39 ^e^	4.38 m; 4.47 m	4.39 m; 4.50 m

*δ* in ppm; *J* in Hz; ^a^ NMR spectra recorded at 400 MHz; ^b^ NMR spectra recorded at 600 MHz; ^c–f^ overlapped signals, assignments may be interchangeable.

**Table 2:** ^13^C NMR spectroscopic data of compounds **1**–**7** (in pyridine-*d*_5_).

Position	1 ^a^	2 ^b^	3 ^b^	4 ^b^	5 ^b^	6 ^b^	7 ^b^
1	38.6	38.5	39.1	39.1	38.3	39.1	38.8
2	27.9	26.6	26.8	26.8	26.6	27.1	26.5
3	89.9	89.8	89.9	89.9	89.9	89.0	89.7
4	40.1	40.1	40.0	40.0	39.9	39.9	39.6
5	55.7	55.7	56.2	56.2	55.5	56.4	55.8
6	18.6	18.5	18.8	18.8	18.6	18.9	18.6
7	32.8	32.6	32.8	33.4	32.8	33.7	33.4
8	41.1	41.1	40.1	40.2	43.4	40.1	40.5
9	54.8	54.8	48.3	48.3	134.6	48.4	47.6
10	37.0	36.9	37.2	37.3	36.7	37.4	36.9
11	127.7	128.4	24.1	24.1	128.6	24.2	24.0
12	130.8	130.4	123.1	123.1	125.8	127.9	128.4
13	142.5	143.9	144.4	144.6	138.6	137.7	139.3
14	41.8	41.7	42.4	42.4	41.6	43.1	42.0
15	26.8	26.7	28.5	28.6	25.4	28.7	29.2
16	27.0	27.9	23.7	23.7	33.4	26.6	26.0
17	48.0	48.0	47.3	47.7	51.8	49.6	48.6
18	129.6	127.0	42.0	41.5	55.4	47.5	54.4
19	212	212.3	46.5	41.2	45.0	37.6	72.6
20	47.8	47.6	31.0	36.7	39.7	153.6	42.1
21	28.5	28.0	34.2	29.1	33.6	28.2	26.6
22	39.3	38.9	33.4	32.3	40.2	31.9	37.7
23	28.4	28.4	28.6	28.6	28.1	28.5	28.3
24	16.5	16.4	17.0	17.0	16.2	17.3	16.9
25	18.3	18.3	15.8	15.8	18.4	16.1	15.6
26	17.1	16.9	17.7	17.8	16.9	17.6	17.3
27	20.4	20.2	26.3	26.3	19.3	26.0	24.6
28	178.9	175.1	176.8	176.8	176.5	176.5	177.3
29	28.2	28.3	33.4	74.0	21.1	21.1	27.0
30	16.6	16.5	23.9	20.0	20.5	113.1	16.7
3-*O*-	Xyl	Xyl	Xyl	Xyl	Xyl	Xyl	2-sulfo-Xyl
1	106.2	106.2	106.1	106.1	106.1	108	104.8
2	79.3	79.7	79.7	79.7	79.0	75.9	80.2
3	78.1	78.2	78.2	78.2	78.0	79.0	77.2
4	71.6	71.6	71.6	71.6	71.4	71.6	70.7
5	67.0	66.9	66.9	67.0	66.8	67.4	65.9
Intermediate	Glc	Glc	Glc	Glc	Glc		
1	102.6	102.5	102.5	102.5	102.2		
2	79.7	79.6	79.6	79.6	79.5		
3	79.4	79.3	79.4	79.4	79.1		
4	72.7	72.9	72.9	73.0	72.4		
5	78.9	79.0	79.2	79.3	78.7		
6	63.6	63.6	63.6	63.6	63.4		
Terminal	Rha	Rha	Rha	Rha	Rha		
1	102.3	102.4	102.3	102.3	102.1		
2	72.9	72.7	72.7	72.7	72.6		
3	73.0	72.9	72.9	72.9	72.7		
4	74.6	74.6	74.6	74.4	74.3		
5	69.8	69.8	69.7	69.8	69.5		
6	19.2	19.2	19.2	19.3	19.0		
28-*O*-		Glc	Glc	Glc	Glc	Glc	Glc
1		95.6	96.0	96.0	96.2	96.3	95.8
2		74.5	74.4	74.7	74.3	74.4	73.9
3		78.9	79.1	79.2	79.4	79.7	78.7
4		71.5	71.3	71.4	71.3	71.4	71.2
5		78.9	78.8	78.8	78.8	79.2	79.1
6		62.6	62.4	62.5	62.3	62.5	62.3

*δ* in ppm; *J* in Hz; ^a^ NMR spectra recorded at 100 MHz; ^b^ NMR spectra recorded at 150 MHz.

## Supplementary Materials

The IR, HRESIMS, and 1D and 2D NMR spectra of the new compounds are available in the supplementary materials.
