# Supplementary material for: Pubescenosides E–K, Seven New Triterpenoid Saponins from the Roots of Ilex pubescens and Their Anti-Inflammatory Activity
Source: Molecules. 2018 Jun 12;23(6):1426. doi: 10.3390/molecules23061426 (PMC6100132; doi:10.3390/molecules23061426)
Supplement: Supplementary file 1 [file molecules-23-01426-s001.zip › molecules-313665-SI/molecules-313665-supplementary/Supporting information.doc]

**Supporting Information**

**Pubescenosides E-K, seven new anti-inflammatory triterpenoid saponins from the roots of *Ilex pubescens***

**Xiaoxu Qiao ^1^, Mengying Ji** **^1^, Yunda Yao ^2^, Leilei Ma ^1^, Jinjun Wu^1^, Guochao Liao ^1^, Hua Zhou ^2^, Zhongqiu Liu ^1,2^ and Peng Wu ^1,^***

^1^ Joint Laboratory for Translational Cancer Research of Chinese Medicine of the Ministry of Education of the People’s Republic of China, International Institute for Translational Chinese Medicine, Guangzhou University of Chinese Medicine, Guangzhou, Guangdong Province, 510006, PR China. qiaoxiaoxu87@126.com (Xiaoxu Qiao); [18826415793@163.com](mailto:18826415793@163.com) (Mengying Ji); maleilei1@126.com (Leilei Ma); wujinjun@gzucm.edu.cn (Jinjun Wu); liao@gzucm.edu.cn (Guochao Liao); liuzq@gzucm.edu.cn (Zhongqiu Liu);

^2^ State Key Laboratory of Quality Research in Chinese Medicine, Macau University of Science and Technology, Taipa, Macau, 999078, PR China. Yunda_Yao@126.com (Yunda Yao); huazhou2009@gmail.com (Hua Zhou).

*Correspondence: wupeng@gzucm.edu.cn (Peng Wu) Tel: +86-20-39358647.

**List of Supporting information**

**Figure S 1.** HRESIMS spectrum of new compound **1**	4

**Figure S 2.** IR spectrum of new compound **1**	4

**Figure S 3.** ^1^H NMR (400MHz, pyridine-*d*_5_) spectrum of new compound **1**	5

**Figure S 4.** ^13^C NMR (100MHz, pyridine-*d*_5_) spectrum of new compound **1**	5

**Figure S 5.** DEPT-135 (100MHz, pyridine-*d*_5_) spectrum of new compound **1**	6

**Figure S 6.** ^1^H-^1^H COSY (400MHz, 400MHz pyridine-*d*_5_) spectrum of new compound **1**	6

**Figure S 7.** HSQC NMR (400MHz, 100MHz pyridine-*d*_5_) spectrum of new compound **1**	7

**Figure S 8.** HMBC NMR (400MHz, 100MHz pyridine-*d*_5_) spectrum of new compound **1**	7

**Figure S 9.** ROESY NMR (400MHz, 400MHz pyridine-*d*_5_) spectrum of new compound **1**	8

**Figure S 10.** HRESIMS spectrum of new compound **2**	8

**Figure S 11.** IR spectrum of new compound **2**	9

**Figure S 12.** ^1^H NMR (600MHz, pyridine-*d*_5_) spectrum of new compound **2**	9

**Figure S 13.** ^13^C NMR (150MHz, pyridine-*d*_5_) spectrum of new compound **2**	9

**Figure S 14.** DEPT-135 (150MHz, pyridine-*d*_5_) spectrum of new compound **2**	10

**Figure S 15.** ^1^H-^1^H COSY (600MHz, 600MHz pyridine-*d*_5_) spectrum of new compound **2**	10

**Figure S 16.** HSQC NMR (600MHz, 150MHz pyridine-*d*_5_) spectrum of new compound **2**	11

**Figure S 17.** HMBC NMR (600MHz, 150MHz pyridine-*d*_5_) spectrum of new compound **2**	11

**Figure S 18.** NOESY NMR (600MHz, 600MHz pyridine-*d*_5_) spectrum of new compound **2**	12

**Figure S 19.** HRESIMS spectrum of new compound **3**	12

**Figure S 20.** IR spectrum of new compound **3**	13

**Figure S 21.** ^1^H NMR (600MHz, pyridine-*d*_5_) spectrum of new compound **3**	13

**Figure S 22.** ^13^C NMR (150MHz, pyridine-*d*_5_) spectrum of new compound **3**	13

**Figure S 23.** DEPT-135 (150MHz, pyridine-*d*_5_) spectrum of new compound **3**	14

**Figure S 24.** ^1^H-^1^H COSY (600MHz, 600MHz pyridine-*d*_5_) spectrum of new compound **3**	14

**Figure S 25.** HSQC NMR (600MHz, 150MHz pyridine-*d*_5_) spectrum of new compound **3**	15

**Figure S 26.** HMBC NMR (600MHz, 150MHz pyridine-*d*_5_) spectrum of new compound **3**	15

**Figure S 27.** NOESY NMR (600MHz, 600MHz pyridine-*d*_5_) spectrum of new compound **3**	16

**Figure S 28.** HRESIMS spectrum of new compound **4**	16

**Figure S 29.** IR spectrum of new compound **4**	17

**Figure S 30.** ^1^H NMR (600MHz, pyridine-*d*_5_) spectrum of new compound **4**	17

**Figure S 31.** ^13^C NMR (150MHz, pyridine-*d*_5_) spectrum of new compound **4**	17

**Figure S 32.** DEPT-135 (150MHz, pyridine-*d*_5_) spectrum of new compound **4**	18

**Figure S 33.** ^1^H-^1^H COSY (600MHz, 600MHz pyridine-*d*_5_) spectrum of new compound **4**	18

**Figure S 34.** HSQC NMR (600MHz, 150MHz pyridine-*d*_5_) spectrum of new compound **4**	19

**Figure S 35.** HMBC NMR (600MHz, 150MHz pyridine-*d*_5_) spectrum of new compound **4**	19

**Figure S 36.** NOESY NMR (600MHz, 600MHz pyridine-*d*_5_) spectrum of new compound **4**	20

**Figure S 37.** HRESIMS spectrum of new compound **5**	20

**Figure S 38.** IR spectrum of new compound **5**	21

**Figure S 39.** ^1^H NMR (600MHz, pyridine-*d*_5_) spectrum of new compound **5**	21

**Figure S 40.** ^13^C NMR (150MHz, pyridine-*d*_5_) spectrum of new compound **5**	21

**Figure S 41.** DEPT-135 (150MHz, pyridine-*d*_5_) spectrum of new compound **5**	22

**Figure S 42.** ^1^H-^1^H COSY (600MHz, 600MHz pyridine-*d*_5_) spectrum of new compound **5**	22

**Figure S 43.** HSQC NMR (600MHz, 150MHz pyridine-*d*_5_) spectrum of new compound **5**	23

**Figure S 44.** HMBC NMR (600MHz, 150MHz pyridine-*d*_5_) spectrum of new compound **5**	23

**Figure S 45.** NOESY NMR (600MHz, 600MHz pyridine-*d*_5_) spectrum of new compound **5**	24

**Figure S 46.** HRESIMS spectrum of new compound **6**	24

**Figure S 47.** IR spectrum of new compound **6**	25

**Figure S 48.** ^1^H NMR (600MHz, pyridine-*d*_5_) spectrum of new compound **6**	25

**Figure S 49.** ^13^C NMR (150MHz, pyridine-*d*_5_) spectrum of new compound **6**	25

**Figure S 50.** DEPT-135 (150MHz, pyridine-*d*_5_) spectrum of new compound **6**	26

**Figure S 51.** ^1^H-^1^H COSY (600MHz, 600MHz pyridine-*d*_5_) spectrum of new compound **6**	26

**Figure S 52.** HSQC NMR (600MHz, 150MHz pyridine-*d*_5_) spectrum of new compound **6**	27

**Figure S 53.** HMBC NMR (600MHz, 150MHz pyridine-*d*_5_) spectrum of new compound **6**	27

**Figure S 54.** NOESY NMR (600MHz, 600MHz pyridine-*d*_5_) spectrum of new compound **6**	28

**Figure S 55.** HRESIMS spectrum of new compound **7**	28

**Figure S 56.** IR spectrum of new compound **7**	29

**Figure S 57.** ^1^H NMR (600MHz, pyridine-*d*_5_) spectrum of new compound **7**	29

**Figure S 58.** ^13^C NMR (150MHz, pyridine-*d*_5_) spectrum of new compound **7**	29

**Figure S 59.** DEPT-135 (150MHz, pyridine-*d*_5_) spectrum of new compound **7**	30

**Figure S 60.** ^1^H-^1^H COSY (600MHz, 600MHz pyridine-*d*_5_) spectrum of new compound **7**	30

**Figure S 61.** HSQC NMR (600MHz, 150MHz pyridine-*d*_5_) spectrum of new compound **7**	31

**Figure S 62.** HMBC NMR (600MHz, 150MHz pyridine-*d*_5_) spectrum of new compound **7**	31

**Figure S 63.** NOESY NMR (600MHz, 600MHz pyridine-*d*_5_) spectrum of new compound **7**	32

**Figure S 1. HRESIMS spectrum of new compound 1**

**Figure S 2. IR spectrum of new compound 1**

**Figure S 3. ^1^H NMR (400MHz, pyridine-d5) spectrum of new compound 1**

**Figure S 4. ^13^C NMR (100MHz, pyridine-d5) spectrum of new compound 1**

**Figure S 5. DEPT-135 (100MHz, pyridine-d5) spectrum of new compound 1**

**Figure S 6. ^1^H-^1^H COSY (400MHz, 400MHz pyridine-d5) spectrum of new compound 1**

**Figure S 7. HSQC NMR (400MHz, 100MHz pyridine-d5) spectrum of new compound 1**

**Figure S 8. HMBC NMR (400MHz, 100MHz pyridine-d5) spectrum of new compound 1**

**Figure S 9. ROESY NMR (400MHz, 400MHz pyridine-d5) spectrum of new compound 1**

**Figure S 10. HRESIMS spectrum of new compound 2**

**Figure S 11. IR spectrum of new compound 2**

**Figure S 12. ^1^H NMR (600MHz, pyridine-d5) spectrum of new compound 2**

**Figure S 13. ^13^C NMR (150MHz, pyridine-d5) spectrum of new compound 2**

**Figure S 14. DEPT-135 (150MHz, pyridine-d5) spectrum of new compound 2**

**Figure S 15. ^1^H-^1^H COSY (600MHz, 600MHz pyridine-d5) spectrum of new compound 2**

**Figure S 16. HSQC NMR (600MHz, 150MHz pyridine-d5) spectrum of new compound 2**

**Figure S 17. HMBC NMR (600MHz, 150MHz pyridine-d5) spectrum of new compound 2**

**Figure S 18. NOESY NMR (600MHz, 600MHz pyridine-d5) spectrum of new compound 2**

**Figure S 19. HRESIMS spectrum of new compound 3**

**Figure S 20. IR spectrum of new compound 3**

**Figure S 21. ^1^H NMR (600MHz, pyridine-d5) spectrum of new compound 3**

**Figure S 22. ^13^C NMR (150MHz, pyridine-d5) spectrum of new compound 3**

**Figure S 23. DEPT-135 (150MHz, pyridine-d5) spectrum of new compound 3**

**Figure S 24. ^1^H-^1^H COSY (600MHz, 600MHz pyridine-d5) spectrum of new compound 3**

**Figure S 25. HSQC NMR (600MHz, 150MHz pyridine-d5) spectrum of new compound 3**

**Figure S 26. HMBC NMR (600MHz, 150MHz pyridine-d5) spectrum of new compound 3**

**Figure S 27. NOESY NMR (600MHz, 600MHz pyridine-d5) spectrum of new compound 3**

**Figure S 28. HRESIMS spectrum of new compound 4**

**Figure S 29. IR spectrum of new compound 4**

**Figure S 30. ^1^H NMR (600MHz, pyridine-d5) spectrum of new compound 4**

**Figure S 31. ^13^C NMR (150MHz, pyridine-d5) spectrum of new compound 4**

**Figure S 32. DEPT-135 (150MHz, pyridine-d5) spectrum of new compound 4**

**Figure S 33. ^1^H-^1^H COSY (600MHz, 600MHz pyridine-d5) spectrum of new compound 4**

**Figure S 34. HSQC NMR (600MHz, 150MHz pyridine-d5) spectrum of new compound 4**

**Figure S 35. HMBC NMR (600MHz, 150MHz pyridine-d5) spectrum of new compound 4**

**Figure S 36. NOESY NMR (600MHz, 600MHz pyridine-d5) spectrum of new compound 4**

**Figure S 37. HRESIMS spectrum of new compound 5**

**Figure S 38. IR spectrum of new compound 5**

**Figure S 39. ^1^H NMR (600MHz, pyridine-d5) spectrum of new compound 5**

**Figure S 40. ^13^C NMR (150MHz, pyridine-d5) spectrum of new compound 5**

**Figure S 41. DEPT-135 (150MHz, pyridine-d5) spectrum of new compound 5**

**Figure S 42. ^1^H-^1^H COSY (600MHz, 600MHz pyridine-d5) spectrum of new compound 5**

**Figure S 43. HSQC NMR (600MHz, 150MHz pyridine-d5) spectrum of new compound 5**

**Figure S 44. HMBC NMR (600MHz, 150MHz pyridine-d5) spectrum of new compound 5**

**Figure S 45. NOESY NMR (600MHz, 600MHz pyridine-d5) spectrum of new compound 5**

**Figure S 46. HRESIMS spectrum of new compound 6**

**Figure S 47. IR spectrum of new compound 6**

**Figure S 48. ^1^H NMR (600MHz, pyridine-d5) spectrum of new compound 6**

**Figure S 49. ^13^C NMR (150MHz, pyridine-d5) spectrum of new compound 6**

**Figure S 50. DEPT-135 (150MHz, pyridine-d5) spectrum of new compound 6**

**Figure S 51. ^1^H-^1^H COSY (600MHz, 600MHz pyridine-d5) spectrum of new compound 6**

**Figure S 52. HSQC NMR (600MHz, 150MHz pyridine-d5) spectrum of new compound 6**

**Figure S 53. HMBC NMR (600MHz, 150MHz pyridine-d5) spectrum of new compound 6**

**Figure S 54. NOESY NMR (600MHz, 600MHz pyridine-d5) spectrum of new compound 6**

**Figure S 55. HRESIMS spectrum of new compound 7**

**Figure S 56. IR spectrum of new compound 7**

**Figure S 57. ^1^H NMR (600MHz, pyridine-d5) spectrum of new compound 7**

**Figure S 58. ^13^C NMR (150MHz, pyridine-d5) spectrum of new compound 7**

**Figure S 59. DEPT-135 (150MHz, pyridine-d5) spectrum of new compound 7**

**Figure S 60. ^1^H-^1^H COSY (600MHz, 600MHz pyridine-d5) spectrum of new compound 7**

**Figure S 61. HSQC NMR (600MHz, 150MHz pyridine-d5) spectrum of new compound 7**

**Figure S 62. HMBC NMR (600MHz, 150MHz pyridine-d5) spectrum of new compound 7**

**Figure S 63. NOESY NMR (600MHz, 600MHz pyridine-d5) spectrum of new compound 7**
