# Supplementary material for: Pubescenosides E–K, Seven New Triterpenoid Saponins from the Roots of Ilex pubescens and Their Anti-Inflammatory Activity
Source: Molecules. 2018 Jun 12;23(6):1426. doi: 10.3390/molecules23061426 (PMC6100132; doi:10.3390/molecules23061426)
Supplement: Supplementary file 1 [file molecules-23-01426-s001.zip › molecules-313665-SI/molecules-313665-supplementary/Tables and figures.doc]

**Tables**

**Table 1.** ^1^H NMR spectroscopic data of compounds **1-7** (in pyridine-*d*_5_).

| **Position** | **1^a^** | **2^b^** | **3^b^** | **4^b^** | **5^b^** | **6^b^** | **7^b^** |
| --- | --- | --- | --- | --- | --- | --- | --- |
| 1 | 0.95 m; 1.77 m | 0.94 m;1.71 m | 0.95 m; 1.50 m | 0.91 m; 1.46 m | 0.97 m; 1.75 m | 0.98 m; 1.48 m | 0.90 m;1.54 m |
| 2 | 1.68 m; 2.56 m | 1.75 m;2.12 m | 1.88 m; 2.06 m | 1.86 m; 2.09 m | 1.96 m; 2.13 m | 1.89 m; 2.17 m | 1.93 m; 2.12 m |
| 3 | 3.30 dd (11.6, 3.7) | 3.28 dd (11.5,4.1) | 3.28 dd (11.4, 3.6) | 3.27 dd (11.4, 4.1) | 3.27 d (10.7, 3.1) | 3.40 d (11.5, 3.8) | 3.29 d (10.8, 2.5) |
| 5 | 0.83 m | 0.79 m | 0.80 m | 0.76 m | 0.76 m | 0.81 m | 0.80 m |
| 6 | 1.33 m; 1.54 m | 1.27 m;1.52 m | 1.30 m; 1.49 m | 1.25 m; 1.44 m | 1.26 m; 1.47 m | 1.27 m; 1.46 m | 1.27 m; 1.45 m |
| 7 | 1.33 m | 1.26 m | 1.46 m | 1.31 m; 1.43 m | 1.16 m | 1.37 m; 1.46 m | 1.40 m; 1.54 m |
| 9 | 2.04 m | 1.98 m | 1.63 m | 1.61 m | － | 1.76 m | 1.78 m |
| 11 | 5.62 d (10.2) | 5.59 d (9.8) | 1.93 m | 1.87 m | 6.58 d (10.1) | 1.91 m; 1.98 m | 2.0 m; 2.06 m |
| 12 | 6.18 d (8.5) | 6.03 d (10.1) | 5.42 s | 5.45 s | 5.61 d (10.6) | 5.48 s | 5.57 s |
| 15 | 1.24 m; 1.92 m | 1.16 m; 1.90 m | 1.17 m; 2.33 m | 1.15 m; 2.32 m | 0.88 m; 1.93 m | 1.17 m; 2.35 m | 1.26 m; 2.44 m |
| 16 | 1.23 m; 2.15 m | 1.30 m; 2.13 m | 1.17 m; 1.26 m | 1.99 m; 2.16 m | 1.64 m; 2.15 m | 1.85 m; 1.99 m | 3.12 m |
| 18 | 5.88s | 5.72s | 3.19 m | 3.28 m | 1.92 m | 3.99 s | 2.92 m |
| 19 | － | － | 1.25 m; 1.75 m | 1.40 m; 2.11 m | 2.30 m | 2.59 m | － |
| 20 | 2.53 m | 2.50 m | － | － | 1.44 m | － | 1.36 m |
| 21 | 1.33 m; 1.66 m | 1.32 m; 1.59 m | 1.08 m; 1.33 m | 1.24 m; 1.74 m | 1.50 m; 1.58 m | 1.28 m; 1.76 m | 2.04 m; 2.12 m |
| 22 | 1.99 m | 1.95 m | 1.75 m; 1.97 m | 1.73 m; 1.84 m | 1.28 m; 2.62 m | 1.76 m; 2.08 m | 1.79 m; 2.04 m |
| 23 | 1.34 s | 1.32 s | 1.33 s | 1.32 s | 1.27 s | 1.31 s | 1.42 s |
| 24 | 1.06 s | 1.03 s | 1.07 s | 1.08 s | 1.04 s | 0.99 s | 1.18 s |
| 25 | 0.82 s | 0.76 s | 0.82 s | 0.83 s | 0.81 s | 0.88 s | 0.89 s |
| 26 | 0.85 s | 0.82 s | 1.07 s | 1.09 s | 0.98 s | 1.09 s | 1.14 s |
| 27 | 1.06 s | 1.03 s | 1.24 s | 1.25 s | 0.99 s | 1.27 s | 1.73 s |
| 29 | 2.11 s | 2.13 s | 0.91 s | 3.56 m | 1.32 d (4.0) | 1.06 d (6.7) | 1.42 s |
| 30 | 1.08 d (8.7) | 1.03 d (7.1) | 0.87 s | 1.08 s | 0.76 d (5.9) | 5.04^c^ | 1.11 d (6.2) |
| 3-O- | Xyl | Xyl | Xyl | Xyl | Xyl | Xyl | 2-sulfo-Xyl |
| 1 | 4.92 d (5.2) | 4.89 d (6.7) | 4.88 d (6.6) | 4.88 d (6.6) | 4.84 d (6.1) | 4.85 d (7.4) | 4.97 d (6.6) |
| 2 | 4.42^e^ | 4.01^c^ | 4.29^d^ | 4.28^d^ | 4.22^d^ | 4.03 m | 5.05 m |
| 3 | 3.87 m | 3.87 m | 3.88 m | 3.86 m | 3.84 m | 4.17 m | 4.43 m |
| 4 | 4.08^c^ | 4.06^c^ | 4.08^c^ | 4.06^c^ | 4.02^c^ | 4.21 m | 4.21 m |
| 5 | 3.73 m; 4.28^d^ | 3.72 m; 4.28^d^ | 3.72 m; 4.28^d^ | 3.72 m; 4.27^d^ | 3.68 m; 4.25^d^ | 3.78 m; 4.38 m | 3.74 m; 4.21 m |
| Intermediate | Glc | Glc | Glc | Glc | Glc |  |  |
| 1 | 5.79 d (5.8) | 5.80 d (7.3) | 5.81 d (7.8) | 5.82 d (7.2) | 5.71 d (6.8) |  |  |
| 2 | 4.24^d^ | 4.27^d^ | 4.24^d^ | 4.22^d^ | 4.22^d^ |  |  |
| 3 | 4.48^e^ | 4.44^e^ | 4.44^c^ | 4.41^e^ | 4.39^e^ |  |  |
| 4 | 4.07^c^ | 4.06^c^ | 4.04^c^ | 4.03^c^ | 3.83 m |  |  |
| 5 | 4.28^d^ | 4.27^d^ | 4.43^e^ | 4.29^d^ | 4.39^e^ |  |  |
| 6 | 4.27^d^; 4.50^e^ | 4.28^d^; 4.51^e^ | 4.28^d^; 4.51^e^ | 4.27^d^; 4.48^e^ | 4.21^d^; 4.48^e^ |  |  |
| Terminal | Rha | Rha | Rha | Rha | Rha |  |  |
| 1 | 6.39 br s | 6.38 br s | 6.39 br s | 6.40 br s | 6.30 br s |  |  |
| 2 | 4.71 m | 4.71 m | 4.75 m | 4.76 m | 4.69 s |  |  |
| 3 | 4.04^c^ | 4.05^c^ | 4.04^c^ | 4.04^c^ | 3.95^c^ |  |  |
| 4 | 4.33^d^ | 4.34^d^ | 4.34^e^ | 4.23^d^ | 4.29^d^ |  |  |
| 5 | 5.03 m | 5.04 m | 5.04 m | 5.04 m | 4.98 m |  |  |
| 6 | 1.81^f^ | 1.78^f^ | 1.79 d (6.0) | 1.8 d (6.0) | 1.75 d (5.5) |  |  |
| 28-O- |  | Glc | Glc | Glc | Glc | Glc | Glc |
| 1 |  | 6.32 d (8.2) | 6.32 d (8.4) | 6.35 d (7.8) | 6.23 d (7.8) | 6.34 d (8.1) | 6.25 d (7.8) |
| 2 |  | 4.48^e^ | 4.21^d^ | 4.33^e^ | 4.13^d^ | 4.23 m | 4.24 m |
| 3 |  | 4.44^e^ | 4.29^d^ | 4.41^e^ | 4.39^e^ | 4.04 m | 4.33^c^ |
| 4 |  | 4.29^d^ | 4.35^e^ | 4.35^e^ | 4.23^d^ | 4.37 m | 4.33^c^ |
| 5 |  | 4.44^e^ | 4.29^d^ | 4.28^d^ | 4.22^d^ | 4.30 m | 4.08 m |
| 6 |  | 4.29^d^ | 4.43^e^ | 4.39^e^; 4.45^e^ | 4.27^d^; 4.39^e^ | 4.38 m; 4.47 m | 4.39 m; 4.50 m |
| *δ* in ppm; *J* in Hz. | | | | | | | |
| ^a^NMR spectra recorded at 400 MHz; ^b^NMR spectra recorded at 600 MHz. | | | | | | | |
| ^c-f^overlapped signals, assignments may be interchangeable. | | | | | | | |

**Table 2.** ^13^C NMR spectroscopic data of compounds **1-7** (in pyridine-*d*_5_).

| **Position** | **1^a^** | **2^b^** | **3^b^** | **4^b^** | **5^b^** | **6^b^** | **7^b^** |
| --- | --- | --- | --- | --- | --- | --- | --- |
| 1 | 38.6 | 38.5 | 39.1 | 39.1 | 38.3 | 39.1 | 38.8 |
| 2 | 27.9 | 26.6 | 26.8 | 26.8 | 26.6 | 27.1 | 26.5 |
| 3 | 89.9 | 89.8 | 89.9 | 89.9 | 89.9 | 89.0 | 89.7 |
| 4 | 40.1 | 40.1 | 40.0 | 40.0 | 39.9 | 39.9 | 39.6 |
| 5 | 55.7 | 55.7 | 56.2 | 56.2 | 55.5 | 56.4 | 55.8 |
| 6 | 18.6 | 18.5 | 18.8 | 18.8 | 18.6 | 18.9 | 18.6 |
| 7 | 32.8 | 32.6 | 32.8 | 33.4 | 32.8 | 33.7 | 33.4 |
| 8 | 41.1 | 41.1 | 40.1 | 40.2 | 43.4 | 40.1 | 40.5 |
| 9 | 54.8 | 54.8 | 48.3 | 48.3 | 134.6 | 48.4 | 47.6 |
| 10 | 37.0 | 36.9 | 37.2 | 37.3 | 36.7 | 37.4 | 36.9 |
| 11 | 127.7 | 128.4 | 24.1 | 24.1 | 128.6 | 24.2 | 24.0 |
| 12 | 130.8 | 130.4 | 123.1 | 123.1 | 125.8 | 127.9 | 128.4 |
| 13 | 142.5 | 143.9 | 144.4 | 144.6 | 138.6 | 137.7 | 139.3 |
| 14 | 41.8 | 41.7 | 42.4 | 42.4 | 41.6 | 43.1 | 42.0 |
| 15 | 26.8 | 26.7 | 28.5 | 28.6 | 25.4 | 28.7 | 29.2 |
| 16 | 27.0 | 27.9 | 23.7 | 23.7 | 33.4 | 26.6 | 26.0 |
| 17 | 48.0 | 48.0 | 47.3 | 47.7 | 51.8 | 49.6 | 48.6 |
| 18 | 129.6 | 127.0 | 42.0 | 41.5 | 55.4 | 47.5 | 54.4 |
| 19 | 212 | 212.3 | 46.5 | 41.2 | 45.0 | 37.6 | 72.6 |
| 20 | 47.8 | 47.6 | 31.0 | 36.7 | 39.7 | 153.6 | 42.1 |
| 21 | 28.5 | 28.0 | 34.2 | 29.1 | 33.6 | 28.2 | 26.6 |
| 22 | 39.3 | 38.9 | 33.4 | 32.3 | 40.2 | 31.9 | 37.7 |
| 23 | 28.4 | 28.4 | 28.6 | 28.6 | 28.1 | 28.5 | 28.3 |
| 24 | 16.5 | 16.4 | 17.0 | 17.0 | 16.2 | 17.3 | 16.9 |
| 25 | 18.3 | 18.3 | 15.8 | 15.8 | 18.4 | 16.1 | 15.6 |
| 26 | 17.1 | 16.9 | 17.7 | 17.8 | 16.9 | 17.6 | 17.3 |
| 27 | 20.4 | 20.2 | 26.3 | 26.3 | 19.3 | 26.0 | 24.6 |
| 28 | 178.9 | 175.1 | 176.8 | 176.8 | 176.5 | 176.5 | 177.3 |
| 29 | 28.2 | 28.3 | 33.4 | 74.0 | 21.1 | 21.1 | 27.0 |
| 30 | 16.6 | 16.5 | 23.9 | 20.0 | 20.5 | 113.1 | 16.7 |
| 3-O- | Xyl | Xyl | Xyl | Xyl | Xyl | Xyl | 2-sulfo-Xyl |
| 1 | 106.2 | 106.2 | 106.1 | 106.1 | 106.1 | 108 | 104.8 |
| 2 | 79.3 | 79.7 | 79.7 | 79.7 | 79.0 | 75.9 | 80.2 |
| 3 | 78.1 | 78.2 | 78.2 | 78.2 | 78.0 | 79.0 | 77.2 |
| 4 | 71.6 | 71.6 | 71.6 | 71.6 | 71.4 | 71.6 | 70.7 |
| 5 | 67.0 | 66.9 | 66.9 | 67.0 | 66.8 | 67.4 | 65.9 |
| Intermediate | Glc | Glc | Glc | Glc | Glc |  |  |
| 1 | 102.6 | 102.5 | 102.5 | 102.5 | 102.2 |  |  |
| 2 | 79.7 | 79.6 | 79.6 | 79.6 | 79.5 |  |  |
| 3 | 79.4 | 79.3 | 79.4 | 79.4 | 79.1 |  |  |
| 4 | 72.7 | 72.9 | 72.9 | 73.0 | 72.4 |  |  |
| 5 | 78.9 | 79.0 | 79.2 | 79.3 | 78.7 |  |  |
| 6 | 63.6 | 63.6 | 63.6 | 63.6 | 63.4 |  |  |
| Terminal | Rha | Rha | Rha | Rha | Rha |  |  |
| 1 | 102.3 | 102.4 | 102.3 | 102.3 | 102.1 |  |  |
| 2 | 72.9 | 72.7 | 72.7 | 72.7 | 72.6 |  |  |
| 3 | 73.0 | 72.9 | 72.9 | 72.9 | 72.7 |  |  |
| 4 | 74.6 | 74.6 | 74.6 | 74.4 | 74.3 |  |  |
| 5 | 69.8 | 69.8 | 69.7 | 69.8 | 69.5 |  |  |
| 6 | 19.2 | 19.2 | 19.2 | 19.3 | 19.0 |  |  |
| 28-O- |  | Glc | Glc | Glc | Glc | Glc | Glc |
| 1 |  | 95.6 | 96.0 | 96.0 | 96.2 | 96.3 | 95.8 |
| 2 |  | 74.5 | 74.4 | 74.7 | 74.3 | 74.4 | 73.9 |
| 3 |  | 78.9 | 79.1 | 79.2 | 79.4 | 79.7 | 78.7 |
| 4 |  | 71.5 | 71.3 | 71.4 | 71.3 | 71.4 | 71.2 |
| 5 |  | 78.9 | 78.8 | 78.8 | 78.8 | 79.2 | 79.1 |
| 6 |  | 62.6 | 62.4 | 62.5 | 62.3 | 62.5 | 62.3 |
| *δ* in ppm; *J* in Hz. | | | | | | | |
| ^a^NMR spectra recorded at 100 MHz; ^b^NMR spectra recorded at 150 MHz | | | | | | | |

**Figures**

**Fig. 1.** Structures of compounds **1-10**.

**Fig.2.** Key HMBC correlations of compound **1**

**Fig.3.** Key ROESY correlations of compound **1**

**Fig.4.** Effect of compounds 1-10 on iNOS and COX-2 protein expression in LPS-stimulated RAW264.7 cells. RAW264.7 cells were seeded in 24-well plate and cultured overnight. Subsequently, compounds 1-10 and the positive control DEX were administered to RAW264.7 cells for 1 h, and LPS (100 ng/ml) was used to stimulate the cells for the final 18 h. Western blotting was performed to analyze protein expression of iNOS, COX-2, and the corresponding values were quantitated by Odyssey v3.0 software. The results are expressed as the mean±SEM, n=3. * P<0.05, ** P<0.01, *** P<0.001, vs LPS group.
